# Community health and medical provision: impact on neonates (the CHAMPION trial)

**DOI:** 10.1186/1471-2431-7-26

**Published:** 2007-07-12

**Authors:** Peter Boone, Vera Mann, Alexander Eble, Tarana Mendiratta, Rohini Mukherjee, Ryan Figueiredo, Chitra Jayanty, Chris Frost, M Reddy Padmanabh, Diana Elbourne

**Affiliations:** 1 Effective Intervention, Centre for Economic Performance, London School of Economics, Houghton Street, London, UK; 2 Medical Statistics Unit, London School of Hygiene and Tropical Medicine, Keppel Street, London, UK; 3 Global Partnerships, Naandi Foundation, 502, Trendset Towers, Road 2, Banjara Hills, Hyderabad – 500034, India; 4 NICE Foundation, 4-1-1056, Bogulkunta, Abids, Hyderabad – 500001, India

## Abstract

**Background:**

The trial aims to evaluate whether neonatal mortality can be reduced through systemic changes to the provision and promotion of healthcare. Neonatal mortality rates in India are high compared to other low income countries, and there is a wide variation of rates across regions. There is evidence that relatively inexpensive interventions may be able to prevent up to 75% of these deaths. One area with a particularly high rate is Mahabubnagar District in Andhra Pradesh, where neonatal mortality is estimated to be in the region of 4–9%. The area suffers from a vicious cycle of both poor supply of and small demand for health care services. The trial will assess whether a package of interventions to facilitate systemic changes to the provision and promotion of healthcare may be able to substantially reduce neonatal mortality in this area and be cost-effective. If successful, the trial is designed so that it should be possible to substantially scale up the project in regions with similarly high neonatal mortality throughout Andhra Pradesh and elsewhere.

**Methods/Design:**

This trial will be a cluster-randomised controlled trial involving 464 villages in Mahabubnagar District. The package of interventions will first be introduced in half of the villages with the others serving as controls. The trial will run for a period of three years. The intervention in the trial has two key elements: a community health promotion campaign and a system to contract out healthcare to non-public institutions. The health promotion campaign will include a health education campaign, participatory discussion groups, training of village health workers and midwives, and improved coordination of antenatal services. The intervention group will also have subsidised access to pregnancy-related healthcare services at non-public lth centres (NPHCs).

The primary outcome of the trial will be neonatal mortality. Secondary outcomes will include age at and cause of neonatal death, neonatal morbidity, maternal mortality and morbidity, health service usage, costs and several process and knowledge outcomes.

**Discussion:**

The trial will be run by independent research and service delivery arms and supervised by a trial steering committee. A data monitoring committee will be put in place to monitor the trial and recommend stopping/continuation according to a Peto-Haybittle rule. The primary publication for the trial will follow CONSORT guidelines for cluster randomised controlled trials. Criteria for authorship of all papers, presentations and reports resulting from the study will conform to ICMJE standards.

**Trial Registration:**

Current Controlled Trials ISRCTN24104646

## Background

### Research justification and relevant literature

Neonatal mortality accounts for almost 40% of all deaths of children under five worldwide, approximately 3.9 million deaths annually [1]. (neonatal mortality and other terms of interest are defined in Table 1 in the appendix) The *Lancet Neonatal Survival Series *asserts that a very small group of causes – including severe infection, prematurity, and birth asphyxia/childbirth complications – lead to the vast majority of these deaths [2]. These and the other major causes of neonatal mortality result in three million newborn deaths that "could be prevented annually by improving access to the low-cost, low-tech interventions that are not currently reaching those most in need," [3]. Our trial tests whether a functional, cost-effective and scalable programme, based on interventions suggested by this literature, can reduce neonatal mortality.

**Table 1:** Glossary of terms

Neonatal Mortality Rate	Number of deaths during the first 28 completed days of life per 1000 live births in a given year.
Perinatal Mortality Rate	The number of perinatal deaths per 1000 total births in a given year. The perinatal period starts at the beginning of foetal viability (28 weeks gestation) and ends at the end of the 7th day after delivery. Perinatal deaths are the sum of stillbirths plus early neonatal deaths.
Infant Mortality Rate	Number of children dying under a year of age per 1000 live births in a given year.
Village Health Worker	A local woman from the village trained primarily to deliver health education to eligible women and refer them to the midwife for antenatal pregnancy-related and postnatal services.
Gram Panchayat	The Gram Panchayat is the executive organ of the Gram Sabha, an agency for planning and development at the village level. Its size varies from 15–30, and the population it covers also varies widely from 5,000 – 15,000. The members of the Panchayat hold office for 3–4 years. Every Panchayat has an elected President (Sarpanch) and the Vice president (Panchayat Secretary) The powers and functions of the Panchayat Secretary are very wide – they cover the entire field of civic administration, including sanitation and public health and of social and economic development in the villages.
Thanda	Administratively, a thanda refers to a hamlet with a small population (around 100 households) vis-à-vis 'villages'. Each thanda is considered a part of a nearby village although the two habitations could be distant from each other.
Penta	A Tribal Chenchu village is known as a "penta". Each penta consists of few huts that are spaced apart and are grouped together based on kinship pattern. The close relatives live nearby and the distant ones farther away. Their homes comprise of few belongings and are generally sparse and Spartan in appearance.
Anganwadi Worker	Angan literally means "courtyard." Under the Integrated Child Development Services Scheme, there is an Anganwadi worker for each population of 1000 people. She is selected from the community and undergoes training in various aspects of health, nutrition and child development. She is a part time worker and is paid an honorarium for service including health checkups, immunisation, supplementary nutrition, health education, and informal preschool education and referral services for women in general and children below the age of 6 years.
DALYs	Disability Adjusted Life Years is the sum of years of potential life lost due to premature mortality and the years of productive life lost due to disability.
Gram Sabha	An assembly of all the adults of a given village which meets at least twice in a year. The Gram Sabha considers proposals for taxation, discusses annual programmes and elects members of the Gram Panchayat.

The methods for implementing this programme are also derived from the public health literature. There is a growing body of evidence showing the potential efficacy of community interventions to address public health problems and disseminate health knowledge, however there exists a large research gap in the literature evaluating such interventions [4-7,3]. Similarly, contracting out of health service provision to non-public enterprises appears promising, yet is also under-researched [8-11]. Our study looks to investigate the potential of these two types of intervention in tandem and in the context of the Lancet Neonatal Survival Series findings [2].

The multi-faceted approach the trial adopts is also rooted in the literature on public health interventions. McLeroy et al. emphasize the need for public health interventions to approach health problems on social and environmental levels, as well as at the individual level, in order to obtain significant results [12]. Others have suggested that multi-faceted health interventions have greater likelihood of success than those which target individuals alone [13,14].

Aside from the obvious motivation of rapidly and significantly reducing neonatal mortality, these interventions also have great promise in their sustainability and knock-on effects. Investments in maternal education are largely recognized to have long-term positive effects on child survival [15]. Similarly, there is evidence that infant morbidity due to infection is significantly related to lifelong disease-related impairment [16,17]. Improvement in maternal education and reduction of infant morbidity as a result of our intervention may yield long-term benefits beyond the time-scale of the trial.

### Background information on the trial area

Neonatal mortality constitutes 65% of all infant deaths in India [18]. In the last three decades, consistent efforts have been underway to reduce child mortality by emphasizing universal immunization, oral rehydration therapy and control of respiratory tract infections. While infant mortality in India has declined from 129 (per 1000 live births) in 1970 to about 68 in 2000, the fate of neonates has remained virtually unaltered: one out of 22 infants in India does not reach the second month of life [19,20].

The state of Andhra Pradesh reports an aggregate neonatal mortality rate (NMR) of 44 per 1000 live births [20]. There is very little reliable information quantifying neonatal survival rates in rural areas and tribal regions, although these rates are known anecdotally to be even higher.

In 1998, a survey in the Mahabubnagar district of Andhra Pradesh found infant mortality rates (IMR) to be much higher than the national average. Summarised results of this survey are shown in Table 2.

**Table 2:** District and division-level estimates of Infant Mortality Rate with 95% confidence levels in Mahabubnagar district of Andhra Pradesh [20]

**Area**	IMR*	**95% CI**
Mahabubnagar district (overall)	*115*	*107 *– *122*
Gadwal division	93	60 – 127
Mahabubnagar division	110	91 – 128
Narayanpet division	125	102 – 147
Wanarparthy division	62	35 – 89
Nagarkurnool division	140	117 – 163

*Infant mortality rate per 1000 live births

The Nagarkurnool division of Mahabubnagar had an infant mortality rate of 140 per 1000 live births. It is believed that approximately 50% of the infant deaths in this division occur in the first month of life [20]. We have chosen to conduct the trial in the Nagarkurnool division in Mahabubnagar on account of the high mortality rate in the area.

Any strategy to reduce neonatal mortality faces several important obstacles. First, a substantial fraction of births occur in unsafe conditions, usually at home. This reflects longstanding traditions and lack of knowledge regarding safe birth practices. Second, institutional care at public health facilities is often geographically inaccessible. Third, even if distance is not a problem, these facilities also suffer chronically from lack of medical practitioners and insufficient medical supplies. The problem faced is thus threefold: health knowledge, demand for health care, and supply of good quality health care are all insufficient. The trial intervention will augment all of these components in order to assess whether, together, they substantially reduce neonatal mortality rates.

### Objectives

The aim of the trial is to test the hypothesis that a community health promotion campaign, in combination with improved supply of health services through contracting out to the non-public sector, can lead to a substantial reduction in neonatal mortality. The programme is designed to improve health knowledge while enhancing both demand for, and supply of, health care.

An important element of the trial is to evaluate the sustainability of such a programme, its cost effectiveness, and the potential to scale it up to meet needs in other high neonatal mortality regions of Andhra Pradesh and elsewhere.

## Methods/Design

### Endpoints of the study

The primary outcome of the study is neonatal mortality. Secondary outcomes will include age at and cause of neonatal death, neonatal morbidity, maternal mortality and morbidity, health service usage, costs and several process and knowledge outcomes.

We will not be dividing foetal losses into miscarriages or stillbirths for two reasons: firstly, gestational age is not reliably reported; secondly, since women in the intervention arm may report pregnancies earlier than women in the control arm, we anticipate there could be a bias in the measurement of gestational age, and this bias may differ across the two arms of the trial.

### Type of study

The trial is a cluster-randomised controlled trial involving 464 villages, with the village as the primary unit of randomisation. This is an unblinded study as, following randomisation, participants and their caregivers will be aware of whether or not they are in an intervention village or control village. For outcomes such as death, this lack of blinding is unlikely to lead to outcome assessment bias.

### Randomisation

Villages will be stratified according to whether their travel time to the nearest designated NPHC is less or greater than one hour, and also into three groups according to whether the village is thanda (2–3 km from the main village with around 15 families), penta (20–30 km from the main village with around 4–5 families) or non-tribal (a main village). All villages will be randomly allocated, within each of these six strata, to either the intervention or control group.

### Interventions – Intervention group

The trial will attempt to reduce neonatal mortality in the intervention villages through a multi-level program: community health promotion and contracting out primary and secondary health services to non-public providers. All the services described below will be provided free to pregnant women and neonates in the intervention communities.

#### Community Health Promotion

To stimulate both adoption of healthy practices and demand for care, the trial will implement a concerted effort to promote health knowledge of communities in the intervention arm. The three main components of this are as follows:

• Training Village Health Workers and Midwives to deliver antenatal services

The trial will select and train local women in the intervention villages to serve as Village Health Workers (VHWs). The person selected as VHW will usually be an Auxiliary Nurse Midwife (ANM) or Traditional Birth Attendant (TBA) already resident in the village. The person chosen will be the most highly skilled available who is acceptable to the communities in the village.

The VHW will be extensively trained by doctors and other health professionals in domiciliary mother and baby care along with postnatal and infant care. They will also be trained in how to deliver antenatal care and conduct safe deliveries at home. They will make home visits to pregnant women and to mothers with neonates. They will also assist with home deliveries.

Approximately every two weeks, teams of two midwives will visit intervention villages offering a package of antenatal care and services (referred to below as "fixed-day services"). Midwives will be trained by doctors to deliver clear, correct pregnancy and neonate-related health information to mothers and their health care providers. They will be trained in community mobilization and the use of communication tools to address deep seated superstitions and practices that have negative impacts on the health of both mother and child. One midwife will act as the main medium for communication of sound health information to women in intervention villages. The other midwife will deliver antenatal health checks and identify high risk pregnancies. She will also give advice to the women, relatives and friends who accompany them on antenatal health care issues and birth planning.

These midwives will supervise VHWs, offer guidance, and conduct emergency deliveries if they occur during their visits to the villages. In case of complications, the midwife will be responsible for referring the mother/neonate in distress to the next level of care.

Midwives based in NPHCs will organise and oversee transportation for referred women.

The intervention team will train a total of approximately 250 VHWs (at least one for every village, in certain villages we may have to train more than one) and approximately 20 midwives (16 midwives delivering fixed day services in teams of two and 4 midwives based at NPHCs). They will go through a orientation program and training of not less than 10 days. This residential training will have both theoretical and practical components. It will be given by experts in the field of maternal and neonatal health care.

Additionally midwives will be trained to update a trial-specific health card provided to all eligible women in intervention villages. These cards will track the various services each woman in the intervention group has received under the programme, including immunizations, participation in health groups, regular checkups and hospitalisation. The VHWs and midwives will ensure that these cards are filled correctly and on time by the various health care providers. The midwives will also keep a record of the number of visits they have made to the village, the number of women they have met, the number of emergency deliveries with which they have assisted and the number of cases they have referred to the NPHCs. Midwives will also keep records of the nature of care they provide to each mother and child attended.

For reinforcement of messages, the VHWs and midwives will be given a manual to refer to for review. Regular meetings will be held to allow them to share knowledge regarding their work and problems encountered.

• Providing health education through a public information campaign

One of the most important contributors to poor maternal and neonatal health is a lack of good health information. This problem is particularly apparent in the local delivery and infant rearing practices which are often in contradiction of evidence-based good practice.

Health knowledge and awareness will be generated through frequent village meetings and community events. A health education campaign including theatre, films, focus groups and other media will be launched to promote key themes related to maternal and child health in each village. Folk culture in the form of song and dance will also be adapted to convey important messages to communities. All members of the community, not just women eligible for the trial, will be targeted by these campaigns.

As part of this campaign, VHWs will also make one to one contact through home visits with mothers, providing them with information related to their pregnancy and the health status of their child. These visits will be conducted on a periodic basis and will help reinforce good health care and practice in women and children. During fixed-day services, midwives will also answer health related queries that the women may have during the course of her pregnancy.

Midwives will also conduct regular village health meetings during the days on which fixed-day services take place. During these they will facilitate discussions, show films and use posters, flip charts and flash cards with the aim of promoting good health practices during and after pregnancy. The exact nature and duration of communication exposure will be designed by a health communications expert.

• Organising women's participatory discussion groups

As there is strong evidence that participatory women's groups can rapidly facilitate community-wide adoption of hygienic practices, a vital responsibility of the VHWs in intervention villages will be to facilitate good attendance at group discussions on issues related to maternal and neonatal health [6,7].

VHWs will organize eligible women from each village into participatory discussion groups of a maximum size between 15 and 20 individuals, creating multiple groups if necessary to maintain appropriate group size. These groups will meet during the day when fixed-day services take part. The midwives will conduct these approximately 1–2 hour sessions at a time and venue suitable to a majority of the women. Each session will contain ice-breakers, a review of the last meeting's content, presentation of new themes, reinforcement of themes through activities, demonstrations, games and a small assignment. These discussions will also be a forum where women can encourage each other to access the trial services. Finally, the participatory group will also serve as a group that provides valuable feedback about the various interventions.

Content for these sessions will include discussion of risk factors for the baby and mother, safe delivery techniques, causes of any recent deaths of neonates and mothers in nearby regions, and other issues that are important in the villages. The objective of these meetings is to improve mothers' health knowledge, to encourage greater use of fixed day services and other available health services, to encourage safe delivery techniques and delivery of high risk babies at the referral centre, and to give mothers a forum to discuss solutions to important issues in maternal and newborn health. This approach has been shown to be very successful in a trial in Nepal [6].

#### Contracting out primary and secondary health services to non-public institutions

As outlined in the trial justification, the supply of primary health care in the trial area is inadequate. Required improvements to the public sector were estimated to be prohibitively expensive, and evidence suggests the private sector may be able to provide primary care which is superior to that provided by the public, and potentially at a lower cost [11].

To increase supply of health services, the trial's intervention will designate up to 5 NPHCs to provide primary and secondary level care, made accessible through enhanced emergency transportation. NPHCs will be selected which are equipped with sufficient infrastructure and human resources. It is imperative to provide women in the intervention arm with quality services which are otherwise unavailable or inadequate, as it is expected that the educational component of the intervention will lead to greater awareness of the need for health services, and thus greater uptake of these services.

The list of candidate NPHCs will be drawn-up based on professional capability, existing infrastructure, and public perception of the facility (public perception serves as a way to introduce community participation in the selection process and to render the process transparent). Tie-ups with such facilities will initially be for the period of 3 months after which a favourable review would lead to a 6 month renewal of the contract. Any kind of primary or secondary service that an expectant mother would require during her pregnancy and for one month of the life of her baby would be covered by these facilities. By virtue of the contractual agreement, these NPHCs would be expected to provide around-the-clock service, admission to the general ward, food for the patient and one attendant, drugs, and disposables.

This contract will be subject to a per-patient cost ceiling. A rigorous monitoring system will buttress against over-billing and over-provision of services by the NPHCs.

Transportation will not be part of this contracting system, but will be provided directly at the village level for pick-up and drop off of patients at the NPHCs.

A detailed third party assessment of the quality of the services by the NPHCs will be conducted periodically by a committee of health and other professionals.

### Interventions – Control group

As the control villages will not be offered the trial interventions unless and until they are shown to be effective, yet data collection will still be required in these villages, control villages will receive the Naandi Foundation's *Girl Child Support Programme *[21], which is a component of a larger "Ensuring Children Learn Programme" run by the Naandi Foundation in two other states of India. The *Girl Child Support *component will provide academic and material support to girls between the ages of six and nine. This intervention is aimed at creating opportunities for young girls to attend and remain in school.

It is anticipated that these interventions in the control group will have negligible impact on neonatal mortality. The fact that these interventions are being carried out, however, may improve the extent and quality of the data collected in the control villages.

### Duration

The trial will run over a period of three years.

### Enrolment – Trial site(s) and participants

The trial site is the Nagarkurnool division of Mahabubnagar district in the Indian state of Andhra Pradesh. The trial will involve only those villages in the division with a population of less than 2,500 people. This criterion was decided upon in light of the fact that larger villages and towns generally have better access to health services, lower neonatal mortality rates, and therefore less potential to benefit from the interventions. Under this criterion, a total of 464 villages will be eligible for the trial with a total population of approximately 300,000 people.

### Enrolment – Inclusion criteria

A woman is eligible for inclusion in the trial if she satisfies the following criteria: i) she is married and less than 50 years old, ii) she or her husband have not had a family planning operation, iii) she is resident in one of the 464 villages at the time of a baseline survey that is to be carried out immediately prior to randomization, and iv) she meets all conditions for consent outlined in the consent section. Once the baseline survey has been carried out and the eligible women enrolled, the only permitted addition to the trial will be those women who marry into a trial village. Women will not be added or removed from the list as a result of either temporary or permanent migration from the village at which they are initially registered.

Only those children of eligible women whose estimated date of delivery at enumeration is at least six months after randomization will be included in the trial. As training and establishment of services in intervention villages will require time to be achieved, this lag in measurement is necessary to ensure exposure to the intervention.

### Enrolment – Exclusion criteria

Women and their children will be excluded from the trial if the woman in question is unmarried, if she is aged 50 or over at the time of the baseline survey, if she or her husband went through a family planning operation, or if she refuses to participate in the baseline survey.

### Enrolment – Sources/methods of enlisting participants in trial

An enumerator will be identified and trained in each of the 464 villages. After training, this individual will compile a list of all eligible women in their village and carry out a baseline survey of all such women. The starting point for the list of eligible women will be the official list of residents held by the Anganwadi Worker for the village.

### Enrolment – Dealing with migration

The population of the villages eligible for the trial will not remain static over the duration of the trial. Migration will occur for a number of different reasons. These will include permanent migration (which is relatively rare), marriage into a trial village, and temporary migration. As mentioned above, those women who marry into one of the 464 eligible villages during the duration of the trial will be considered eligible for participation in the trial. Temporary migration includes seasonal migration for work, which is very common in the area under scrutiny, and selective migration in order to give birth in the village where the pregnant woman's mother lives. This selective migration is expected to be nearly universal for women expecting their first child, but much more limited for later deliveries. In addition, some women might migrate because of a wish to benefit from the services provided in intervention villages, although it is anticipated that this is very unlikely.

In total we anticipate that around two-thirds of the deliveries of women enrolled in the trial will take place in the village of enumeration. Of the remaining one third, some will move to a village of the same type as their initial village (control or intervention), some will move to a location beyond the scope of the study, and some will move from intervention to control or vice-versa. This migration poses several challenges to the design of, data collection in, and analysis of the trial.

Because it is anticipated that the benefits of the intervention programme will occur throughout pregnancy and not just at the time of delivery, the trial has been designed to compare neonatal mortality rates according to village of residence rather than village at which delivery takes place. This allows each woman's village to be specified prior to randomisation, which reduces the potential for bias. A structural bulwark will ensure that only those women who are resident in intervention villages at the time of enumeration (along with those who marry in) receive the programme of services. Operationally those women who move from a control to an intervention village pose potentially the greatest problem. These women would normally not have subsidised access to non-public health services. If such exclusions have a negative impact on trial activities and morale in particular villages, then exceptional provision of non-public care will be decided upon in a case-by-case basis by the supervisors.

### Enrolment – Losses to follow-up

Data will be collected on each pregnancy at the enumeration village. It is not anticipated that women who do not migrate will voluntarily withdraw from the trial. When a woman migrates this will be documented and data collected when she returns. Every effort will be made to record data from all women who are included at enumeration to ensure that bias due to migration-related loss of observations is kept to a minimum. Women who migrate beyond the scope of the trial will be sought out for information. In case of inability to locate an enumerated woman, we will interview two of her relatives for birth information. Nonetheless, it is recognised that migration will both reduce the observed effect of the intervention and lead to some loss of information. This has been taken into account in the statistical power calculation.

### Consent

For this trial we will employ three tiers of consent: state, Panchayat, and individual.

• At the state level, approval of the protocol has been obtained from the Department of Health & Family Welfare of the government of Andhra Pradesh.

• Consent will also be obtained from each Panchayat in the trial area. The Panchayat is a democratically elected body that governs a small group of villages. This is the smallest unit of government in rural India. In all villages in the trial, consent will be obtained from the Panchayat in the following manner. These local bodies will convene their general body meetings (also called the Gram Sabhas). The protocol of the trial will be explained to the villagers in their local language. Consent will be given in oral form during a Panchayat meeting with written documentation of the approval given by the Panchayat leader. This process of obtaining consent through meetings with approval of the 'guardians' of the clusters is common in trials in which the intervention is delivered at the level of a cluster and it is not possible to obtained for informed consent for randomisation from individuals within the cluster [22].

• Given this process, women who are asked and agree to take part in the enumeration are considered to have given their informed consent to participate in the trial. Women not living in the villages at that point who subsequently fulfil the eligibility criteria will be informed about the study and asked if they agree to be added to the list.

### Sample size calculation

The sample size has been calculated to give 80% statistical power to show a reduction in neonatal mortality that is statistically significant using a conventional 2-sided significance level of 5% if the true effect of the package of interventions is to reduce neonatal mortality by 25%. We assumed 4.38% neonatal mortality in the control villages (and hence 3.29% neonatal mortality in the intervention villages), an average population of 659 per village, a birth rate of 23 per 1000 population (giving rise to an average of 38 births per village over a period of 2.5 years) and an intra-cluster correlation coefficient of 0.00644 [20,6]. Under these assumptions we require 330 villages to detect a 25% reduction in neonatal mortality.

In the trial area, however, women who are expecting their first child usually move back to their mother's village around one to three months before the expected date of delivery. This migration could reduce the measured effect of intervention if women move from control villages to intervention villages and vice-versa. According to the NFHS-2, 36% of all births are first births in Andhra Pradesh. We assume that 90% of these women temporarily migrate and on the basis of this that 8% of control women actually deliver in an intervention village, whilst 24% of intervention women will deliver in non-intervention villages (8% in control group villages and 16% in villages outside of the study area) [20]. Assuming that both groups incur half the benefits of the intervention, the expected neonatal mortality rates in the two groups become 4.34% and 3.42%. Under this scenario 464 villages will still give 80% statistical power to show a reduction in neonatal mortality that is statistically significant at the 5% level.

### Analysis strategies

The main analysis will assess the effect of the interventions on the primary and secondary outcomes. Primary analysis of the outcome(s) will follow the intention to treat principle (i.e. the participants will remain in the group they were randomised to and not analysed according to the interventions actually received), and will account for clustering.

For the primary outcome (neonatal mortality), the relative risk with a 95% confidence interval will be reported, taking appropriate account of clustering. A generalised linear model (without adjustment for covariates) using robust standard error estimates will be used to carry out this analysis. A further analysis will adjust for distance to nearest healthcare centres (public and private) and migration.

For secondary binary outcomes, including neonatal morbidity, maternal mortality and morbidity, and health service usage, relative risks will be estimated in an analogous fashion. For continuous outcomes t-tests with robust SEs will be carried out. ANCOVA (with robust standard errors) will be used where baseline adjustment is required. Bootstrapping will be used for non-normal outcomes and Cox models for survival analysis. A full analysis plan is given in the operations manual.

### Ethical Approval

The protocol has received ethical approval from the institutional review board of the LV Prasad Eye Institute, Hyderabad, India, which is affiliated with the Indian Council of Medical Research(Ethics Reference Number: LEC07002) as well as from the ethics committee of the London School of Hygiene and Tropical Medicine (application number: 5166).

## Discussion

### Administrative Structures – The implementation team

The trial is constructed with independent research and services teams that are responsible for data collection needed to analyze outcomes, and the implementation of the interventions, respectively. The services team will be responsible for implementing the interventions. They will conduct activities to assess the implementation of interventions in their regions, such as collecting survey data, and tracking the use of various health services by pregnant women in villages. The people employed in the research team will have no role in the services component of the trial and vice-versa. Separating service delivery activities, which occur only in the intervention villages, from research activities, which will occur in both control and intervention villages, is a bulwark against contamination of our data for analysis. These groups are described in further detail below.

The *Service Delivery Team *will be responsible for all service delivery activities in the intervention villages. The members of this team include:

a. A *Programme Coordinator *who will head the team.

b. *Village Health Workers (VHWs)*, women who will be responsible for delivering antenatal and health education to the village assigned to her. In her village, the VHW will also track high risk mothers and refer them to the NPHCs.

c. *Midwives *who will support antenatal care at the village level and conduct safe deliveries. They will accompany high risk pregnancy cases to the NPHCs. They will also be responsible for conducting village visits and performing health communication activities.

d. *Programme Supervisors *and *Programme Officers *who will oversee the day-to-day operations and monitor the field staff.

The *Research Team *will track pregnant women and their babies, as well as perform data collection and monitoring, in both intervention and control villages. The members of this team include:

a. A *Research Coordinator *who will lead the team.

b. A *Research Officer *who will validate collected data and monitor the field staff.

c. *Enumerators *in each village who will identify eligible women and track pregnancies.

d. *Data Supervisors *who will monitor the enumerators and obtain detailed follow-ups on pregnancy outcomes (e.g. deliveries, still births, miscarriages and neonatal death).

### Administrative Structures – Trial management

The trial will be supervised by the Trial Steering Committee and the Trial Management Group. Day to day activities will be the responsibility of the Naandi Foundation with support from Effective Intervention and the LSHTM where appropriate.

The Trial Management Group will include a Trial Manager, Trial Programmer, Database Manager and Trial Statistician. This group will meet monthly, either face-to-face and/or by telephone, to ensure the efficient day-to-day running of the trial, to prepare reports for the steering committee, and to organize and service the data monitoring and ethics committee.

### Administrative Structures – Trial Coordinating Centre

The Naandi Foundation will act as the local Trial Coordinating Centre and be responsible for recruitment, implementing interventions, and handling administrative matters once participants are being entered.

### Administrative Structures – Trial Steering Committee

The Trial Steering Committee (TSC) will meet periodically to discuss the overall progress of the trial. It will include all principal investigators, trial manager, one or two experienced investigators not otherwise involved in the trial, and a statistician. The group will meet initially to finalize the protocol and organisation. Once the trial is running, the group will meetregularly to discuss issues such as recruitment progress and protocol deviations from the Data Monitoring Committee. The TSC will evaluate this information and take or refrain from action accordingly.

### Administrative Structures – The Data Monitoring Committee

The Data Monitoring Committee (DMC) will be completely independent of the trial and will meet regularly to review interim results. The principal role of the DMC is to monitor accumulating data by random allocation and alert the organizers of the trial if they think patterns in the data – indicating benefits, hazards or both – are sufficiently persuasive to warrant either closing recruitment to a trial or changing the protocol. The DMC will include a biostatistician and clinicians with expertise in the topic under trial. Data will be given to the DMC on a confidential basis by the trial statistician.

### Interim analyses

Interim analyses will be pre-specified, but may be amended if requested by the DMC. The analyses for the DMC will be performed by the trial statistician only and no other members of the trial research group will have access to the results.

The DMC will first assess the data when data on the outcome of contributing births (i.e. not including births in the first six months post randomisation) from no less than one full year is available. A Peto-Haybittle rule will be utilised to help the DMC to assess whether to recommend that the trial should be stopped for efficacy or harm, but the DMC will also take into account the balance of risks and benefits as well as consistency with external evidence [23,24].

### Surveillance/data collection

In each village, data will be collected by the Enumerator on a monthly basis on paper forms which will then be entered into a database. Data Supervisors will perform follow-up interviews according to data collected by the enumerator, to determine pregnancy outcomes and relevant information which is then reported to the Coordinating Centre. Each pregnancy will be followed until at least six weeks after delivery. The Coordinating Centre will compile the data and perform internal checks for data integrity and consistency.

The trial will use a computerised system for data management to keep track of the flow of participants in the trial as well as for recording the continuous flow of information to and from participants. The system will provide efficient data validation, quality control and standardised management reports.

### Quality control programme

To ensure quality of clinical centres, data handling and processing in the coordinating centre, a quality control programme will be established to run concurrently with the intervention. This will include a system of data checking, audit trails (detailed logs showing data which have been changed, the reason for any change, who made the change and when), and source verification procedures. In the process, information about adverse events, compliance and protocol deviations, contamination, co-interventions, and documentation of losses and dropouts will be systematically recorded.

### Data and safety monitoring

The TSC will ensure the safety of individual patients participating in the trial by setting up and running the trial in accordance with good practice. Neither the TSC nor any other team member involved with running the trial will have sight of accumulating data by allocation during the course of the trial.

### Economic evaluation and sustainability of measures post-intervention

An important goal of the study is to analyze the costs and benefits of the approach, and the sustainability of the outcomes. We will conduct a cost-effectiveness analysis, measured in terms of the cost per disability adjusted life year (DALY) saved, when the trial is completed. In this analysis, we will compare the effects of the trial's intervention with those of other interventions targeting neonatal death.

### Ethics/protection of human subjects – Stopping Guidelines

Guidelines are outlined in the section above describing the data monitoring committee.

### Ethics/protection of human subjects – Research governance and good clinical practice

The trial will comply with all relevant legal and professional standards. These include ethics (especially protecting patient confidentiality); science (ensuring research is well designed); information (ensuring that research findings are published and accessible); health and safety (ensuring the safety of staff and participants); and finance (being accountable for spending research funds).

### Ethics/protection of human subjects – HIV/AIDS

In both arms of the trial, mothers/women who appear tobe at high risk for HIV/AIDS will be advised toget counselled and tested atdesignated government VCTCs (voluntary counselling and testing centres). The trial team will facilitate easy accessto these centres by providing transport and liaison with professionals at the centres.

### Publication policy – Reporting and dissemination

Prior to their submission or application for presentation, all manuscripts, posters or oral presentations, and other reports of the outcomes of this research effort will be approved by a majority of the TSC. All publications will include a formal acknowledgement that the trial was designed and executed by the LSHTM, the Naandi Foundation, the NICE Foundation, and Effective Intervention, and that financial support was provided by Effective Intervention. The primary report of the trial will follow the reporting guidelines in the CONSORT Statement for cluster RCTs [25].

### Publication policy – Authorship

The authorship of manuscripts, posters, oral presentations, and any other reports of the results of this study will be guided by the criteria for authorship formulated by the International Committee of Medical Journal Editors as published in its "Uniform Requirements for Manuscripts Submitted to Biomedical Journals" [26]. According to these requirements, the authors should meet the following criteria: each author should have participated sufficiently in the work to take public responsibility for the content. Authorship credit should be based only on substantial contributions to (a) conception and design, or analysis and interpretation of data; and to (b) drafting the article or revising it critically for important intellectual content; and on (c) final approval of the version to be published. Conditions (a), (b), and (c) must all be met.

As a general, but not absolute, rule, at least one individual from each of the four partner organizations – the Naandi Foundation, Effective Intervention, the NICE Foundation and the LSHTM – will be authors for all publications that result from this research.

## Abbreviations

ANCOVA Analysis of Covariance

ANM Auxiliary Nurse Midwife

DALY Disability Adjusted Life Year

DMC Data Monitoring Committee

IMR Infant Mortality Rate

ISRCTN International Standard Randomised Controlled Trial Number

LSHTM London School of Hygiene and Tropical Medicine

NICE Neonatal Intensive Care and Emergencies

NMR Neonatal Mortality Rate

NPHC Non Public Health Centres

RCT Randomised Controlled Trial

TBA Traditional Birth Attendant

TSC Trial Steering Committee

VHW Village Health Worker

## Competing interests

The Naandi Foundation and the NICE Foundation are together actively involved in health programs intervening with women and children in rural and urban Andhra Pradesh and Rajasthan. Those authors not affiliated with the Naandi Foundation have no competing interests to declare.

## Authors' contributions

PB conceived the study, participated in its design and obtained funding. DE, CF and VM participated in study design and the statistical analyses. CJ and TM participated in conceiving the study and design. RP participated in conceiving the study and contributed to designing the service delivery arm. RM, RF and AE participated in study design. All authors have read and approved the manuscript.

## Pre-publication history

The pre-publication history for this paper can be accessed here:



## References

[B1] Black RE, Morris SS, Bryce J (2003). Where and why are 10 million children dying every year?. The Lancet.

[B2] Lawn JE, Cousens S, Zupan J (2005). 4 million neonatal deaths: When? Where? Why?. The Lancet.

[B3] Bhutta ZA, Darmstadt GL, Darmstadt, Hasan BS, Haws RA (2005). Community-Based Interventions for Improving Perinatal and Neonatal Health Outcomes in Developing Countries: A Review of the Evidence. Pediatrics.

[B4] Bhandari N, Bahl R, Mazumdar S, Martines J, Black R, Bhan M (2003). Effect of community-based promotion of exclusive breastfeeding on diarrhoeal illness and growth: a cluster randomised controlled trial. The Lancet.

[B5] Haider R, Ashworth A, Kabir I, Huttly SRA (2000). Effect of community-based peer counsellors on exclusive breastfeeding practices in Dhaka, Bangladesh: a randomised controlled trial. The Lancet.

[B6] Manandhar DS, Osrin D, Shrestha N, Mesko J, Morrison K, Tumbahangphe K, Tamang S, Thapa S, Shretsha D, Thapa B (2004). Effect of a participatory intervention with women's groups on birth outcomes in Nepal: cluster-randomised controlled trial. The Lancet.

[B7] Paulson S, Gisbert ME, Quiton M (1996). Case Studies of Two Women's Health Projects in Bolivia. Family Health International.

[B8] Bhushan I, Keller S, Schwartz B (2002). Achieving the twin objectives of efficiency and equity. Contracting health services in Cambodia. Asian Development Bank Economics and Research Department Policy Brief Series.

[B9] Liu X, Hotchkiss DR, Bose S, Bitra R, Giedion U (2004). Contracting for Primary Health Services: Evidence on its effects and a framework for evaluation. Technical Consultation on Health Sector Reform and Reproductive Health: Developing the Evidence Base 30th November–2nd December Geneva.

[B10] McPake B, Banda EEN (1994). Contracting out of health services in developing countries. Health Policy Plan.

[B11] Loevinsohn B, Harding A (2005). Buying results? Contracting for health service delivery in developing countries. The Lancet.

[B12] McLeroy KR, Bibeau D, Steckler A, Glanz K (1988). An ecological perspective on health promotion programs. Health Education Quarterly.

[B13] Sorensen G, Emmons K, Hunt M, Johnston D (1998). Implications of the results of community intervention trials. Annual Review of Public Health.

[B14] Green LW, Kreuter MW (1990). Health promotion as a public healthstrategy for the 1990s. Annual Review of Public health.

[B15] Belli PC, Flavia B, Preker A (2005). Investing in children's health: what are the economic benefits?. Bull World Health Organ.

[B16] Niehaus M, Moore SR, Patrick PD (2002). Early childhood diarrhea is associated with diminished cognitive function 4–7 years later in children in a northeast Brazilian shantytown. American Journal of Tropical Medicine and Hygiene.

[B17] Lorntz B, Soares AM, Moore SR, Pinkerton R, Gansneder B, Bovbjerg VE, Guyatt H, Lima AM, Guerrant RL (2006). Early childhood diarrhea predicts impaired school performance. The Pediatric Infectious Disease Journa.

[B18] James KS, Aitken I, Subramanian SV (2000). Neonatal Mortality in India – Emerging Paradoxes. Harvard Center for Population and Development Studies Working Paper Series.

[B19] Claeson M, Bos ER, Mawji T, Pathmanathan I (2000). Reducing child mortality in India in the new millennium. Bull World Health Organ.

[B20] IIPS (International Institute for Population Sciences), ORC Macro (2000). National Family Health Survey (NFHS-2): India.

[B21] Naandi Girl Child Support Scheme. http://www.naandi.org/Support/GirlChild.asp.

[B22] Edwards S, Braunholtz D, Lilford R, Stevens A (1999). Ethical issues in the design and conduct of cluster randomised controlled trials. BMJ.

[B23] Haybittle JL (1971). Repeated assessment of results in clinical trials of cancer treatment. British Journal of Radiology.

[B24] Peto R, Pike MC, Armitage P, Breslow NE, Cox DR, Howard SV, Mantel N, McPherson K, Peto J, Smith PG (1976). Design and analysis of randomized clinical trials requiring prolonged observation of each patient. I. Introduction and design. British Journal of Cancer.

[B25] Campbell MK, Elbourne DR, Altman DG (2004). CONSORT statement: extension to cluster randomised trials. BMJ.

[B26] Davidoff F, Godlee F, Hoey J, Glass R, Overbeke J, Utiger R, Nicholls MG, Horton R, Nylenna M, Hojgaard L, Kotzin S (2003). Uniform requirements for manuscripts submitted to biomedical journals. Journal of the American Osteopathic Association.

